# The Relationship between Tests of Neurocognition and Performance on a Laparoscopic Simulator

**DOI:** 10.1155/2010/486174

**Published:** 2010-06-24

**Authors:** Oumar Kuzbari, Howard Crystal, Pedram Bral, Rima A. A. Atiah, Imad Kuzbari, Amine Khachani, Muhammad Faisal Aslam, Howard Minkoff

**Affiliations:** ^1^Department of Obstetrics and Gynecology, Maimonides Medical Center, 4802 Tenth avenue, Brooklyn, NY 11219, USA; ^2^Department of Neurology, SUNY Downstate Medical Center, Brooklyn, NY 11203, USA

## Abstract

*Objective*. To estimate if there is a relationship between the results of tests of neurocognition and performance on a laparoscopic surgery simulator. *Methods and Materials*. Twenty participants with no prior laparoscopic experience had baseline cognitive tests administered (Trail Making Test, Part A and B (TMT-A and TMT-B), Grooved Peg Board Test, Symbol Digit Modalities Test, Symbol Digit Recall Test, and Stroop Interference Test), completed a demographic questionnaire, and then performed laparoscopy using a simulator. We correlated the results of cognitive tests with laparoscopic surgical performance. *Results*. One cognitive test sensitive to frontal lobe function, TMT-A, significantly correlated with laparoscopic surgical performance on the simulator (correlation coefficient of 0.534 with *P* < .05). However, the correlation between performance and other cognitive tests (TMT-B, Grooved Peg Board Test, Symbol Digit Modalities Test, Symbol Digit Recall Test, and Stroop Interference Test) was not statistically significant. *Conclusion*. Laparoscopic performance may be related to measures of frontal lobe function. Neurocognitive tests may predict motor skills abilities and performance on laparoscopic simulator.

## 1. Introduction

Laparoscopic surgery has become an increasingly important component of the gynecologist's armamentarium. While several factors such as sleep deprivation [1, 2] and substance abuse [3] have been shown to effect abilities with this modality, determinants of skill among rested, sober trainees have not been as clearly delineated. 

 Neurocognition is an important factor in all learning. Neurocognitive enhancement of surgeons through nonpharmacological and psychopharmacological methods has been the subject of recent media, political, and ethical interest [4] A large number of tests of neurocognition, each of which is focused on a different aspect of brain function, have been validated. The frontal brain in particular might be expected to play a role in laparoscopy because of its executive and motor functions that are established through extensive cortical and subcortical connections. Therefore, the following study was undertaken to assess the degree to which tests of neurocognition correlate with learning on laparoscopic simulators.

## 2. Materials and Methods

### 2.1. Population

This was a cohort study of individuals at Maimonides Medical Center (MMC) who had no prior laparoscopic experience, who underwent tests of neurocognition and then performed a task on a laparoscopic simulator. Twenty volunteers (nineteen third year medical students and one midwife) who had no prior laparoscopic experience were invited to participate in our study during their OB/GYN rotation and each gave informed consent. The first twenty participants asked to participate all agreed. The study was approved by the institutional review board.

### 2.2. Materials

#### 2.2.1. Tests of Neurocognition

Trail Making Tests (TMT): These tests consist of two paper-and-pencil challenges, TMT-A, and TMT-B. TMT-A test consists of connecting, in ascending sequential order, 25 numbered circular targets arranged randomly in a paper space without lifting the pencil. TMT-B test consists of linking 23 circular targets, which are divided into a set of numbers (1–13) and set of letters (A-L). In TMT-B, the set of numbers and set of letters must be alternately linked in ascending order: from A-1 to L-13 without lifting the pencil from the paper. The performance of each part of the test is based on the time in seconds needed to complete each part, and on penalizing errors by adding additional time to the final score. 

TMTs are the most commonly used test of neurocognition to assess the executive function of the frontal lobe of the brain [5–7]. It measures the attention, visual scanning, cognitive flexibility, visuospatial sequencing, and speed motor movements [5, 6].

Stroop Interference Test: the Stroop interference test consists of 3 card subtests. The first card contains color words (red, green, and blue) printed in black ink. The second card contains blocks of colors (red, green, and blue). The third card contains the word of the first page printed in the color of the blocks of the second page though and all the colors and words do not match. All three subtests are organized into 10 columns and 6 rows of words. In the first one, the participant reads as many words as possible going down the columns from left to right. In the second subtest, the subject will name as many color blocks as possible. In the third, the participant tries to read every printed color. The score represents the time needed to name correctly all the items from each subtest. If an error is made the subject is redirected and penalized with additional time added to their score. The Stroop interference measures frontal lobe function especially selective attention, cognitive flexibility, information processing speed, and executive function [6, 7].

The Grooved Peg Board Test: using the dominant hand, subjects place asymmetrical metal pegs into 25 key shaped holes in the grooved pegboard while being timed. Once completed the test is repeated with the nondominant hand. The score is based on the length of time necessary to insert all the pins and on the number of pins dropped. The test assesses the speed of fine motor control, eye-hand coordination, and manual dexterity [6, 7].

Symbol Digit Modalities Test: subjects are shown 9 symbols, each with a corresponding letter or number. Subjects are then asked to translate a document with these codes. Participants are given 90 seconds to translate as many symbols as possible. It is designed to measure of cognitive psychomotor speed, visual scanning, and tracking [6, 7].

Symbol Digit Recall Test: participants are shown the 9 digit-symbol pairs for a determined time. Then, they are asked to reproduce the reference key given only the symbol part. The Digit Recall test is a common measure of short-term memory [6, 7]. 

#### 2.2.2. Laparoscopic Simulator Task

**Key Trainer:**

**Figure 1:**
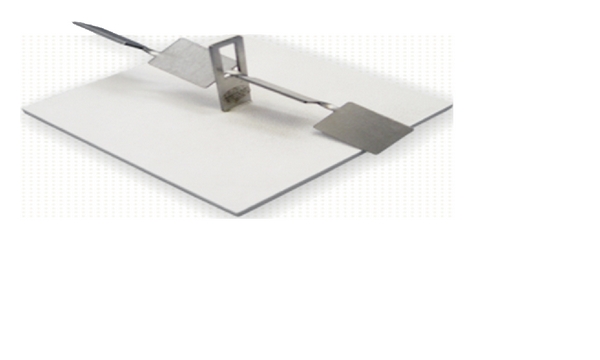
Key trainer

The participants had to perform one LapTrainer SimuVision simulator task to measure their baseline laparoscopic proficiency. The lap Trainer key test is an essential part of the laparoscopic curriculum used to teach basic laparoscopic skills using a laparoscopic simulator [8]. The participants had to use two laparoscopic graspers to pass a specially designed key through a narrow slot in one direction.  Figure 1. The slot was placed at a 45-degree angle to the trainer. The task was designed to replicate the level of motor demands of laparoscopic procedures. The performance is graded based on completion time.

### 2.3. Methods

Each subjects completed a demographic questionnaire. They also completed the Stanford Sleepiness Scale (SSS) and the Positive Affect Negative Affect Scale (PANAS) questionnaires. The Stanford Sleepiness Scale is a common scale to assess sleepiness or alertness at a specific moment in time. The SSS questionnaire required participants to rate their present degree of sleepiness, rated on seven-point scale [9]. The PANAS provides reliable, precise, and largely independent measures of Positive Affect and Negative Affect. It measures their general and specific emotions right before starting the experiment, which comprises ten positive and ten negative mood-related adjectives, rated on a scale of 1 to 5 [10]. After filling in the questionnaires, each subject underwent the above five neurocognitive tests, each of which focused on a different aspect of brain function. The cognitive tests were administered uniformly, in the same order to each participant to avoid variation between participants based on the sequence of tests performed. Then, each participant completed one trial of the laparoscopic surgery task: the Key test.

### 2.4. Data Analysis

#### 2.4.1. Data from the Questionnaires Are Reported as Median and Ranges

The dependent variable analyzed was the Key test. The independent variables examined were mood, the Sanford Sleep Scale and the neurocognitive tests.

Spearman's correlation was used to correlate mood, sleep and neurocognitive test scores with laparoscopic performance to establish if there is any relationship between cognitive abilities and basic motor skills used in laparoscopy. *P* values less than .05 were considered statistically significant. In regard to power, we used the Trail Making Tests to guide our sample size consideration, and anticipated an effect size similar to that seen in the literature for other outcomes linked to TMT [11]. In this case, expecting that an increase in the time to completion of TMT would correlate with a 60% increase in the time to perform the simulation, we calculated that with 20 patients we would have an 80% power to detect that difference.

## 3. Results

A total of 20 volunteers participated in the study. Nine (45%) were female and 11(55%) male. Median and ranges for Sleeping Scale, Positive and Negative PANAS Scale are summarized in Table 1. The median score on the Sanford Sleeping Scale was 2; most of the subjects were awake, responsive and able to concentrate. On the Positive PANAS scale, the median score was 28, while on the Negative PANAS scale the score was 12. Overall, the median Positive PANAS was higher than the median Negative PANAS; participants had stronger positive affects than negative affects.

The Table 2 shows the correlation between the laparoscopic performance and the Sleep and Mood Scales Table 2. No significant correlation was found between the Positive PANAS score or the Negative PANAS and basic motor skills. Similarly, there was no significant correlation between sleep scale and performance on laparoscopy. This may be because participants were not at the extremes on either scale. 

TMT-A, which is a neurocognitive test measuring the function of the frontal lobe, showed significant correlation with the performance on the laparoscopic simulator Table 3. A correlation coefficient of 0.534 was found between the scores on TMT-A and performance on the simulator (*P  *< .05); a high score on TMT-A was associated with a high performance score on simulated surgery. While the TMT-B also showed a strong positive correlation (the more time required to complete the neurocognitive task the greater the time to complete the laparoscopic task), with a correlation coefficient of 0.443, this correlation has approximated significance at traditional levels (*P* = .0503).

The Symbol Digit Number and the Symbol Digit Recall tests had a negative correlation with performance on the simulator which means a high score (a greater number) of translated symbols in a timed interval on these tests correlated with increased performance on simulator (less time to complete a task), but that association was not statistically significant 

The correlation between performance and other cognitive tests (Grooved Peg Board test and Stroop Interference Test) was not statistically significant. (*P *> .05).

## 4. Discussion

Several researchers have investigated the effect of sleep deprivation [1, 2], fatigue [2], and cognitive distraction [12] on laparoscopic surgical performance [8, 13–16]. However, relatively little attention has been paid to determinants of motor and cognitive function, although laparoscopy is complex surgery that involves both functions [17, 18]. Neuropsychologists have generally believed that the frontal lobe of the brain mediates the most complex behavioral and cognitive functions, [19] and it has been linked to planning, attention, sequencing, concentration, and future-oriented thinking [20]. 

Previous studies have validated the use of computerized simulators to evaluate laparoscopic surgical performance [8, 13–16] and many studies have used simulators to establish consequences of fatigue on psychomotor and cognitive decision making skills [1, 2]. Additionally, some basic measures of cognitive ability such as class rankings and USMLE scores have been used to predict baseline laparoscopic abilities during residency training [13]. However, more detailed studies correlating basic laparoscopic skills with tests of neurocognitive function are lacking. The purpose of our study was to analyze the correlation between the results of tests of neurocognition, especially those measuring the function of the frontal lobe, with basic laparoscopic skills. Our study results indicate that neurocognition correlates with operative skills. It also supports findings from previous studies and elucidates potential research areas. 

TMT-A showed a significant correlation with the basic motor skills on the Laptrainer. This test measures frontal lobe function, particularly motor speed, eye hand coordination, attention, concentration, tracking, and the ability to maintain focus. We also found a strong correlation between TMT-B and performance on the LapTrainer with approximated significance at traditional levels (*P *= .0503). 

Functional Magnetic Resonance Imaging (fMRI) offers some insights into what the TMT results actually reflect. fMRI was used to assess brain activation while participants performed the TMT by comparing brain metabolic activities when subjects execute TMT-A compared to TMT-B [5]. TMT-A particularly assesses visual scanning and visuospatial sequencing, while TMT-B also assesses cognitive set shifting [21, 22]. The fMRI findings agreed with the existing literature showing sensitivity of the TMT to frontal regions and found considerable brain activity outside the frontal lobe that differed for TMT-B versus TMT-A [5]. TMT-B engages the middle temporal gyrus and superior temporal gyrus of the left hemisphere supposedly associated with the working memory component of the TMT [5]. Working memory is essential for multitasking and guiding actions toward achievement [6]. However, in our study, the short-term memory test was not significantly correlated with operative skills.

 Laparoscopic performance has been associated with abilities in visuospatial sequencing and visuospatial scanning. These abilities may take place in the connections between the parietal lobe and the frontal lobe. Initially, a visual stimulus triggers the premotor cortex located in the frontal lobe to program grasping movement sequences [20]. A visuomotor loop results from the connection between premotor cortex and anterior intraparietal cortex; it prepares prehension of the instruments [20]. Coordinating movement strategies are controlled at the lateral anterior intraparietal sulcus projecting into the premotor and supplementary motor areas [20, 23].

Caffeine, a psychostimulant, has been reported to improve cognitive task performance and to inhibit delayed reaction time during sleep times of sleep deprivation [24, 25]. Consumers of caffeine also tend to have better performance especially on TMTs, visual speed information processing tasks and visual reaction time [24, 25], and therefore caffeine may stimulate the function of the frontal lobe and improve executive function. 

 Sequential learning is a key in performance improvement. The prefrontal region operates with reciprocal cortical connections and subcortical loops through the thalamus and basal ganglia [7, 26]. Two independent loops within the basal ganglia have been shown to control learning motor skills: the associative/anterior premotor loop and the posterior sensorimotor loop [26]. Early learning of new basic laparoscopic skills may engage the associative corticobasal ganglia loop, whereas advanced operative skills may engage the posterior sensorimotor-basal ganglia loop [26]. 

 On the other hand, consistency in laparoscopic performance may be based on automatization of basic surgical skills; this automatization has been seen in experienced surgeons. It allowed multitasking and blocked the influences of distraction and fatigue on motor skills and cognitive tasks [12, 17, 27]. During learning, automatization was achieved when a dynamic shift of activation occurred from the associative-premotor to the sensorimotor territories of the striatopallidal complex [26]. Mastered motor skills might be stored in the sensorimotor-basal ganglia to sustain the newly automated skills and to enhance execution speed [26]. 

An understanding of basic laparoscopic skills that are associated with tests of neurocognition may help to facilitate a greater understanding of the brain pathways involved in surgical proficiency. Baseline operative skills may be predicted by neurocognition tests [28], which may evaluate the time and training necessary to reach proficiency rather than predicting which candidates will ultimately make proficient surgeons [28]. 

We need to acknowledge a few limitations. Our power was inadequate to assess all functions. However, the number of subjects tested was similar to the number used in other papers, [14, 16, 17, 28] and allowed us to find significance in the most robust relationships. Second, we also only used naïve subjects. Thus, we cannot comment on neurocognitive effects on senior surgeons. Further research is necessary to determine whether such these tests could be helpful as an assessment tool for assessing acquired laparoscopic skills in surgical residency program. However our goal was to look at neurocognition at a time of learning. Third, we tested our subjects on only one simulated laparoscopic task. Using additional tasks of simulated laparoscopy such as the peg transfer task or scoring tasks using additional criteria such as economy of movements and errors could have been considered [1, 8, 28]. However, we chose to evaluate basic operative skills on participants with no prior laparoscopic experience. Multiple laparoscopic tasks may have displaced basic motor skills assessment. Finally it must be recognized that specific neurocognitive tests, though focused on a given part of the brain, may engage many other parts as well. It would be inappropriate therefore to think that TMT-A, for example, solely reflects frontal lobe function. Also, the neurocognitive tests that correlated with laparoscopic skills were timed tests. Thus, the ability to function under time pressure may independently link scores on those tests with laparoscopic skills.

## 5. Conclusion

In conclusion, neurocognitive tests provide insight into brain functions that are involved in laparoscopic performance. It appears that performance is related, at least in part, to the prefrontal lobe where motor abilities are elaborated. That region of the brain has multiple cortical and subcortical connections which are able to interfere with operative skills. Tests of neurocognition appear to provide a global assessment of potential motor skill abilities, which may in turn predetermine laparoscopic performance. Further studies using different tests of cognition, coupled with fMRI, may expand our understanding of this relationship, and provide a more precise understanding of the brain's control of laparoscopic skills.

## Figures and Tables

**Table 1:** Median values for age, sleeping scale and PANAS scale.

	**N**	Minimum	Maximum	Median
Sleeping Scale	20	1	5	2
Positive PANAS Scale	20	14	42	28
Negative PANAS Scale	20	10	29	12

**Table 2:** Impact of sleep and mood on laparoscopic performance.

		Sleeping Scale	PANAS Scale Positive	PANAS Scale Negative
Lap Simulator Task time	Spearman correlation	−.399	.160	− .364
	*P*-value	.080	.498	.114
	*N*	20	20	20

**Table 3:** The relationship between neurocognitive tests and laparoscopic simulator performance.

		Lap Simulator Task Time
TMT-A Time	Spearman Correlation	.534*
	*P*-value	**.015**
	*N*	20

TMT-B Time	Spearman Correlation	**.443**
	*P-*value	**.0503**
	*N*	20

Dominant Hand Grooved Peg Board Time	Spearman Correlation	.242
	*P*-value	.304
	*N*	20

None Dominant Hand Grooved Peg Board Time	Spearman Correlation	.325
	*P*-value	.161
	*N*	20

Symbol Digit Task Number	Spearman Correlation	−.184
	*P*-value	.435
	*N*	20

Symbol Digit Recall Number	Spearman Correlation	−.111
	*P*-value	.639
	*N*	20

Stroop Interference Time	Spearman Correlation	.314
	*P*-value	.176
	*N*	20
